# Migraine attacks during menses: efficacy of eletriptan and relationship to recurrence after response

**DOI:** 10.1186/1129-2377-14-S1-P196

**Published:** 2013-02-21

**Authors:** Almas Bhambri, Chatterjee Abdulsattar

**Affiliations:** 1 Pfizer Inc., USA

## Introduction

Compared with migraine attacks not related to menses, migraine attacks during menstruation have been shown to have longer duration and higher recurrence rates, are more resistant to treatment, and are associated with greater functional disability.

## Objective

To compare migraine attacks during menses to other migraine attacks and assess recurrence of migraine after response in both these groups.

## Design/methods

Data for eletriptan 20 mg(E20), 40 mg(E40), 80 mg(E80) were pooled from 5 similarly designed RCT's of eletriptan. Women with migraine beginning within 1 to 4 days of menstrual flow (Gl) were compared to women with migraine not associated with menses (G2). Headache response within 2 hours to Eletriptan and placebo was compared in women within Gl and G2 using logistic regression analyses controlling for baseline headache severity, treatment group(E20, E40, E80, Pbo) and study. Recurrence of migraine after initial response within 2 hours and up to 24 hours post-headache was compared between Gl and G2 also using logistic regression. Adverse event frequencies were also compared between groups.

## Results

Five studies (N= 3217) were included in this analysis. 2796 (86.9%) of the subjects were women. Mean age for Gl (N= 630) was 36.8 (50= 8.1) and G2 (N= 1586) was 37.7 (50= 9.9) years. Headache response within 2 hours was superior in the Eletriptan treated groups vs. placebo in Gl (E20: OR=3.8, 95% CI=1.8, 7.7; p<0.0002)(E40:OR= 5.3, 95%CI=3.3, 8.7; p<0.0001)(E80: OR=6.5, 95% CI=3.8, 11.05; p< 0.0001) and in G2 (E20: OR= 1.65,95%CI= 1.01,2.7; p<0.045)(E40: OR= 2.9, 95%CI=2.2,3.9; p<0.0001)(E80:OR=4.2, 95%CI=3.1,5.8;p<0.0001). Headache recurrence rates were higher in Gl compared to G2 (OR=1.66, 95%CI=1.22,2.26; p<0.00l). Adverse events were comparable between Gl and G2.

## Conclusions

Eletriptan has similar efficacy in the treatment of migraines occurring within and without a menstrual period. Migraine attacks during menses have higher odds of recurrence than migraines occurring at other times. Eletriptan was safe and well tolerated.

